# Impact of assumptions on future costs, disutility and mortality in cost-effectiveness analysis; a model exploration

**DOI:** 10.1371/journal.pone.0253893

**Published:** 2021-07-12

**Authors:** Amir-Houshang Omidvari, Iris Lansdorp-Vogelaar, Harry J. de Koning, Reinier G. S. Meester

**Affiliations:** Department of Public Health, Erasmus MC University Medical Center Rotterdam, Rotterdam, The Netherlands; Massachusetts General Hospital, UNITED STATES

## Abstract

**Introduction:**

In cost-effectiveness analyses, the future costs, disutility and mortality from alternative causes of morbidity are often not completely taken into account. We explored the impact of different assumed values for each of these factors on the cost-effectiveness of screening for colorectal cancer (CRC) and esophageal adenocarcinoma (EAC).

**Methods:**

Twenty different CRC screening strategies and two EAC screening strategies were evaluated using microsimulation. Average health-related expenses, disutility and mortality by age for the U.S. general population were estimated using surveys and lifetables. First, we evaluated strategies under default assumptions, with average mortality, and no accounting for health-related costs and disutility. Then, we varied costs, disutility and mortality between 100% and 150% of the estimated population averages, with 125% as the best estimate. Primary outcome was the incremental cost per quality-adjusted life-year (QALY) gained among efficient strategies.

**Results:**

The set of efficient strategies was robust to assumptions on future costs, disutility and mortality from other causes of morbidity. However, the incremental cost per QALY gained increased with higher assumed values. For example, for CRC, the ratio for the recommended strategy increased from $15,600 with default assumptions, to $32,600 with average assumption levels, $61,100 with 25% increased levels, and $111,100 with 50% increased levels. Similarly, for EAC, the incremental costs per QALY gained for the recommended EAC screening strategy increased from $106,300 with default assumptions to $198,300 with 50% increased assumptions. In sensitivity analyses without discounting or including only above-average expenses, the impact of assumptions was relatively smaller, but best estimates of the cost per QALY gained remained substantially higher than default estimates.

**Conclusions:**

Assumptions on future costs, utility and mortality from other causes of morbidity substantially impact cost-effectiveness outcomes of cancer screening. More empiric evidence and consensus are needed to guide assumptions in future analyses.

## Introduction

Recommendations for cost-effectiveness analysis in health and medicine suggest to account for the health-related costs and quality of life during the life-years gained (LYG) by an intervention [1]. Health-related expenses, (dis)utility values, and mortality are all affected by both the condition primarily targeted by the intervention, and other causes of morbidity. Cost-effectiveness analyses are more meaningful when realistically representing these effects.

Cancer screening is an important field of application for cost-effectiveness analyses [1]. A complication is that cancer has many shared risk factors with other conditions [2], suggesting that patients in whom cancer is prevented may be at increased risk of other conditions. For example, screening for lung cancer in average-risk populations will likely decrease the risk of death from lung cancer [3]. However, those who benefit from lung cancer screening are mostly smokers with a relatively high risk of other conditions, with implications for the future LYG, quality of life, and costs.

Scant direct data sources exist to inform the future costs, disutility and mortality assumptions specific to patients who benefit from cancer screening. A common assumption is that those who benefit are similar to those invited (i.e. the broader population) in terms of overall risk of morbidity and mortality. In general, future costs and disutilities for other conditions are often not completely taken into account, with unclear implications. For example, modeling suggested the National Health Service breast cancer screening program to be marginally cost-effective [4], but accounting for all future costs might have changed these conclusions.

In this study, we used an established microsimulation model to further investigate the potential impact of different assumptions for the costs, disutility and mortality from other causes of morbidity in two case examples: screening for colorectal cancer (CRC) in the general population, and screening for esophageal adenocarcinoma (EAC) in people with gastroesophageal reflux disease (GERD) symptoms.

## Methods

We used the Microsimulation Screening Analysis (MISCAN) model to estimate the cost-effectiveness of a selection of previously evaluated screening strategies for CRC and EAC under a range of assumptions for the risk of other conditions. For the purposes of this illustration, costs were expressed in US dollar and considered from a healthcare sector perspective, including all health-related expenses. (S1 Table in S1 Appendix). All costs were updated to the year 2020 using the US consumer price index and both costs and effects were discounted at an annual rate of 3%.

### Microsimulation modeling

MISCAN was developed by the Department of Public Health, Erasmus MC University Medical Center Rotterdam, and is being used as part of the Cancer Intervention and Surveillance Modeling Network (CISNET). The model has previously informed cancer screening recommendation by the United States (US) Preventive Services Task Force and the American Cancer Society [5, 6], and has been described in detail elsewhere [7–10]. MISCAN simulates the relevant life histories for a synthetic population reflective of the US population, or a subset of the population. First, a date of birth and date of death for each individual is simulated based on US life tables [11]. During their lifetime, individuals may develop cancer through a number of diseases states, from healthy, through benign precursor lesions (small, medium, or large adenoma for CRC, and Barrett’s esophagus (BE) with no, low-grade, or high-grade dysplasia for EAC), through preclinical cancer, to clinical (symptomatic) cancer (S1 Fig in S1 Appendix). A person may die from clinical cancer, or from other causes first. Screening may lead to gains in LY through detection and treatment of cancer in an earlier stage, or through detection and treatment of precancerous lesion.

### Screening strategies

We re-evaluated a number of strategies considered before in studies from 2016 and 2017 [5, 12]. For CRC, we simulated an average-risk US population cohort aged 40 years, without diagnosed cancer. For EAC, we simulated a 60-year-old US male population with GERD symptoms. First, we followed both cohorts until death in the absence of screening (natural history scenario). Then, for CRC, 20 unique colonoscopy screening strategies were evaluated with varying start ages (45, 55, 65), stopping ages (75, 80, 85), and intervals (5, 10, 15). Patients with detected adenomas received colonoscopy surveillance in 3–5 years depending on risk characteristics, as suggested by US guidelines [13]. For EAC, we evaluated two different screening methods: once-only screening at age 60 years with either endoscopy or cytosponge with endoscopy follow-up of positive cytosponge results [12]. Patients with detected BE entered surveillance or received endoscopic therapy in accordance with guidelines [14].

### Cost-effectiveness definition

The cost-effectiveness ratio was defined as incremental costs over incremental Quality-Adjusted Life-Years (QALYs) of one strategy (strategy 1) *vs*. another (strategy 2). The numerator consists of the incremental costs related to cancer and other causes of morbidity. QALYs represent the value assigned to the duration of life modified by the impairments, functional states and perceptions as influenced by disease, injury or treatment [15]. These can be decomposed into the life-years gained minus the disutility associated with cancer and other causes of morbidity. The components for the costs and disutility from other causes of morbidity were the key focus of the present study, in addition to the other-cause mortality rates behind the life-years lived. We estimated the costs and disutility by assigning age-dependent costs and utility values to each life-year lived, and estimated the impact of different mortality assumptions by scaling individuals’ life expectancy.

### Costs, quality of life, and mortality assumptions

The assumed consumption costs and disutility for cancer screening and treatment [16, 17], and the cause-specific survival were similar to previous analyses (S2 Table in S1 Appendix) [18], and were independent from assumptions for other causes of morbidity. The assumed costs, disutility, and mortality related to other conditions were informed by survey data, life tables, and reports of standardized incidence or standardized mortality rates (SMRs) for (pre)cancerous patients. Specifically, average health-related expenses by age for the U.S. were informed by 2018 Medical Expenditure Panel Survey data [19]. Average health-related quality of life by age was derived by averaging across different measures compared in the study by Hanmer et al. [20] As in previous analyses, average all-cause mortality was informed by U.S. life tables [11]. Continuous cost and disutility estimates by age were derived using weighted nonlinear least squares regression, with age-specific population size as the weights. For patients with precancerous lesions or cancer, it was assumed that the costs, disutility and mortality are proportional to the population averages by a factor representing the overall relative risk of other conditions. A best estimate of the relative risk was derived from various clinical and epidemiological studies. For CRC, an analysis of linked Surveillance Epidemiology and End Results(SEER)-Medicare claims data, reported for 137,536 CRC patients diagnosed between 1995–2002, a relative risk of 1.28 vs. non-cancer patients for having ≥1 comorbidity, and of 1.43 for ≥2 comorbidities [21]. In another study, an SMR of 1.20 (95%CI, 1.18–1.22) was reported for a population-based cohort of 40,826 patients with adenomas removed between 1997–2011 [22]. For EAC, an SMR of 1.24 (95%CI, 1.18–1.31) was reported for a community-based cohort of 8,929 patients with Barrett’s esophagus diagnosed between 1995–2012, excluding deaths from esophageal cancer [23]. While a best estimate of 1.25 for the relative risk of morbidity follows from these data, we varied the relative risk to show the impact of different assumptions.

**Fig 1:**
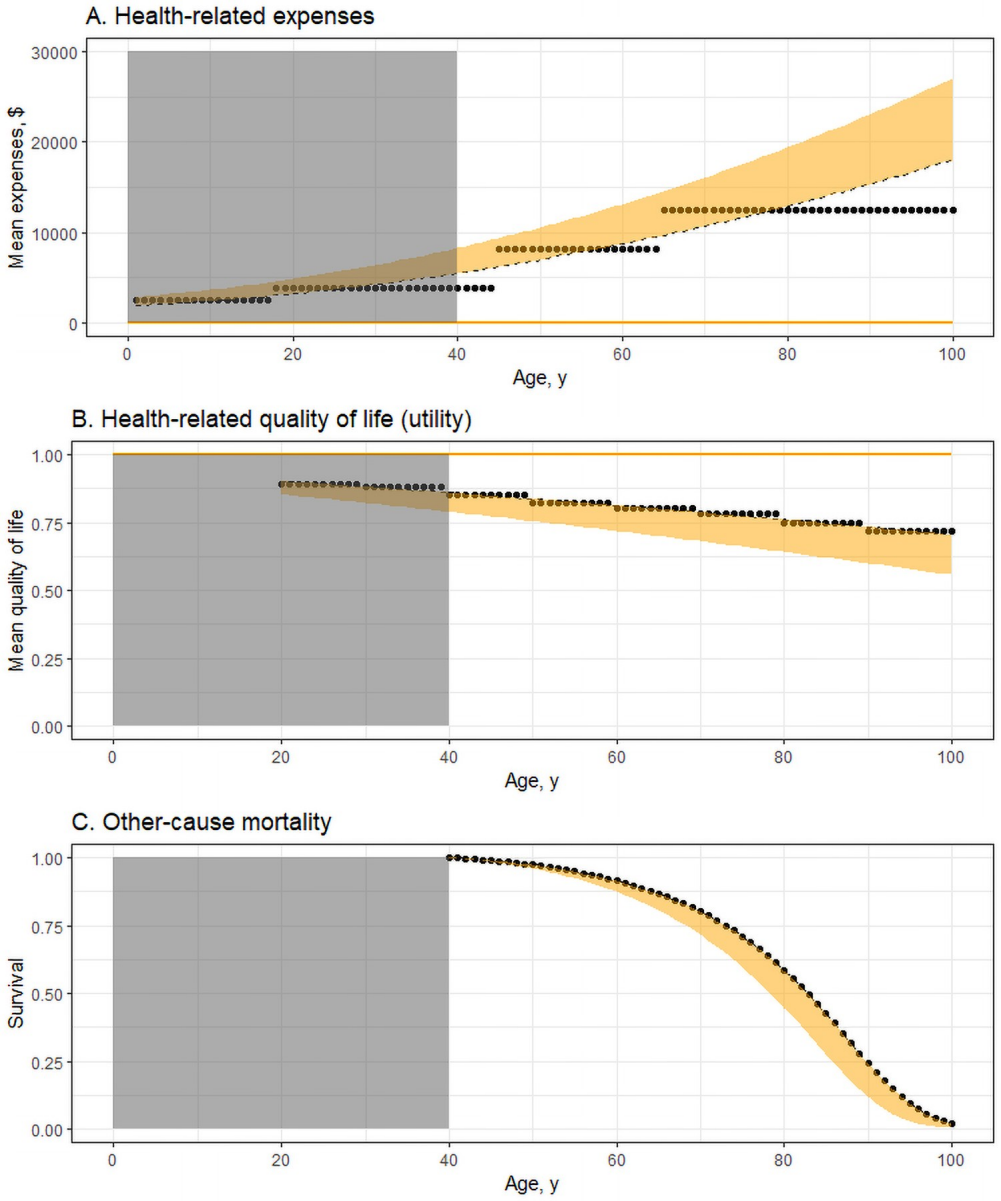
Model assumptions on health-related expenses, quality of life, and mortality by age. ^a^ a Dots represent observed data; black dashed lines fitted using weighted nonlinear least squares regression; yellow ranges alternative assumptions evaluated in this study (incl. zero costs and disutility). Observed data in Panel A are estimated health-related expenses by age from the Medical Expenditure Panel Survey [19]. Observed data in Panel B estimate the health-related quality of life (utility) [20]. Survival rates in Panel C are derived from U.S. life tables.

The main assumptions are summarized in Fig 1.

### Analysis

First, we evaluated the outcomes for the currently recommended screening strategies for CRC: colonoscopy every 10 years from age 50 through 75 years and endoscopic screening for EAC at age 60 years, as recommended for GERD patients with multiple risk factors. Outcomes considered were the discounted LYG, QALY gained, cost and the average cost-effectiveness ratio (ACER), with no screening as the comparator (strategy 2 in the cost-effectiveness definition) [24]. Outcomes were evaluated under different assumptions for the future health-related costs, disutility and mortality. In a default scenario, we assumed average other-cause mortality, and did not account for disutility and costs from conditions other than CRC or EAC. In additional scenarios, we evaluated U.S. average costs, disutility and mortality multiplied by the assumed relative risk of other conditions among precancerous patients, considering values of 1, 1.125, 1.25, 1.375. and 1.5 to investigate the dose-response relationship with outcomes.

Subsequently, we compared the outcomes of all evaluated screening strategies. We assessed incremental cost-effectiveness ratios (ICERs) [25]. ICERs were assessed only for the efficient options among all evaluated strategies, i.e. strategies on the efficient frontier of QALY gained for any given level of expenses, and were assessed relative to the next less effective strategy on the efficient frontier. Again, we estimated the ratios under default assumptions, and average health-related costs and effects multiplied by different assumed relative risks of other causes of morbidity.

In sensitivity analyses, analyses were repeated assuming an annual discount rate of 0% instead of 3%. Also, we repeated analyses including only the excess health-related expenses for precancerous patients *vs*. average adults, instead of total health-related expenses, given average expenses may be partly compensated by future premiums or taxes received, from the perspective of the healthcare payer.

## Results

### Average cost-effectiveness analysis

**Fig 2:**
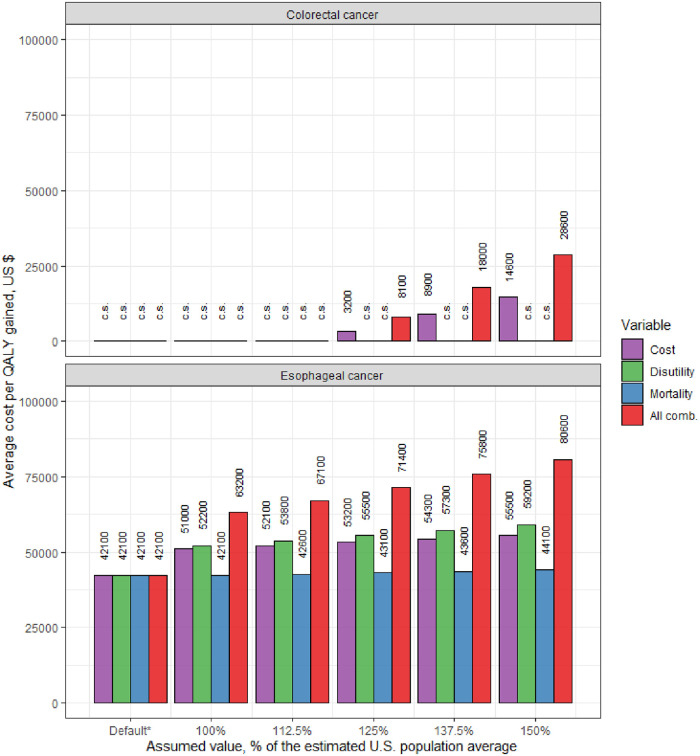
Impact of various assumptions for the health-related costs, disutility and mortality on the average cost-effectiveness of recommended screening strategies for colorectal cancer^a^ and esophageal adenocarcinoma. ^b,c*^ Abbreviations: comb. = combined; c.s.: cost-saving. ^a^ Colonoscopy every 10y from age 50 through 75; ^b^ Endoscopy at age 60 in men with gastroesophageal reflux symptoms. ^c^ Figure shows independent and combined effects of the costs, disutility and mortality from other causes of morbidity (Fig 1) on the cost per QALY gained *vs*. no screening. The X-axis represents the assumed costs, disutility and mortality for precancerous patients relative to the general population. * Default scenario with average mortality, without health-related costs and disutility.

For CRC, under default assumptions of average other-cause mortality and no costs and disutility for conditions other than CRC, the recommended colonoscopy screening every 10 years between age 50 and 75 years was predicted to result in 83 QALY gained per 1000 adults, and net cost-savings of $1.66 million compared to no screening. Fig 2 shows the ACER for the recommended strategy under variable health-related costs, disutility, mortality, and all those factors combined. While higher assumed values for the health-related disutility or mortality alone decreased the QALY gained compared with no screening, this did not change the estimate that screening was cost-saving. However, for health-related costs ≥125% of the U.S. population average, the recommended strategy was no longer cost-saving, and the ACER increased up to $14,600 per QALY gained. By affecting both the denominator and numerator of the ACER, the joint impact of all factors combined exceeded the sum of individual effects, increasing the ACER up to $28,600 per QALY gained.

For EAC, default assumptions of average other-cause mortality and no costs and disutility for conditions other than EAC, endoscopy screening was predicted to result in 32 QALY gained per 1000 men with GERD symptoms, at a net cost of $1.62 million (Table 1), yielding an ACER of $42,100 per QALY gained. Among the assumed costs, disutility and mortality, the assumed disutility had the greatest independent impact on the ACER, increasing it up to $59,200 (Fig 2). As for CRC, the joint impact of all factors was superadditive of the independent effects, increasing the ACER up to $80,600 per QALY gained.

**Table 1:** Cost and effects of colorectal cancer (CRC) and esophageal adenocarcinoma (EAC) screening incremental to no screening, under various assumed values for the future health-related costs, disutility and mortality, per 1,000 adults.

	Assumed health-related costs, disutility and mortality, as % of estimated U.S. population average															
	Default values ^b^				100%				125%				150%			
Strategy ^a^	Cost, $ mln	LY	QALY	ICER, $ ^c^	Cost, $ mln	LY	QALY	ICER, $^c^	Cost, $ mln	LY	QALY	ICER, $^c^	Cost, $ mln	LY	QALY	ICER, $^c^
Colorectal cancer																
**No screening**	Ref.	Ref.	Ref.	Dom.	Ref.	Ref.	Ref.	Dom.	Ref.	Ref.	Ref.	Dom.	**Ref.**	**Ref.**	**Ref.**	**Ref.**
**Colonoscopy, 55–75, 15**	**-1.81**	**64.7**	**70.5**	**Ref.**	**-0.98**	**64.7**	**55.1**	**Ref.**	**-0.06**	**54.7**	**42.9**	**Ref.**	**0.55**	**47.0**	**33.7**	**16,400**
Colonoscopy, 55–85, 15	-1.75	65.0	70.4	Dom.	-0.91	65.0	55.0	Dom.	-0.00	54.9	42.7	Dom.	0.59	47.2	33.5	Dom.
**Colonoscopy, 55–75, 10**	**-1.80**	**68.1**	**73.5**	**3,300**	**-0.92**	**68.1**	**57.3**	**25,100**	0.07	57.5	44.4	Dom.	0.72	49.5	34.8	Dom.
Colonoscopy, 55–85, 10	-1.75	68.3	73.3	Dom.	-0.87	68.3	57.1	Dom.	0.12	57.7	44.2	Dom.	0.76	49.6	34.6	Dom.
Colonoscopy, 55–75, 5	-1.51	71.8	75.3	Dom.	-0.59	71.8	58.2	Dom.	0.49	60.8	44.7	Dom.	1.19	52.3	34.6	Dom.
Colonoscopy, 55–80, 5	-1.46	72.2	75.2	Dom.	-0.53	72.2	58.0	Dom.	0.55	61.0	44.5	Dom.	1.25	52.5	34.4	Dom.
Colonoscopy, 55–85, 5	-1.41	72.2	74.9	Dom.	-0.47	72.2	57.7	Dom.	0.59	61.0	44.2	Dom.	1.29	52.5	34.2	Dom.
**Colonoscopy, 50–75, 15**	-1.67	71.4	77.4	Dom.	-0.77	71.4	60.6	Dom.	**0.22**	**61.1**	**47.8**	**57,400**	**0.90**	**53.2**	**38.0**	**80,800**
Colonoscopy, 50–80, 15	-1.64	72.7	78.1	Dom.	-0.72	72.7	61.1	Dom.	0.28	62.0	47.9	Dom.	0.95	53.9	37.9	Dom.
**Colonoscopy, 50–75, 10** ^d^	**-1.66**	**77.3**	**82.9**	**15,600**	**-0.68**	**77.3**	**64.7**	**32,600**	**0.41**	**66.2**	**50.8**	**61,100**	**1.15**	**57.6**	**40.3**	**111,100**
Colonoscopy, 50–80, 10	-1.62	78.1	83.1	Dom.	-0.63	78.1	64.8	Dom.	0.47	66.7	50.7	Dom.	1.21	58.0	40.1	Dom.
Colonoscopy, 50–75, 5	-1.15	82.6	84.9	Dom.	-0.11	82.6	65.6	Dom.	1.09	70.7	50.8	Dom.	1.91	61.6	39.7	Dom.
Colonoscopy, 50–80, 5	-1.09	82.9	84.8	Dom.	-0.48	82.9	65.3	Dom.	1.16	71.0	50.6	Dom.	1.97	61.8	39.4	Dom.
Colonoscopy, 50–85, 5	-1.04	83.0	84.5	Dom.	0.00	83.0	65.1	Dom.	1.20	71.0	50.3	Dom.	2.00	61.8	39.2	Dom.
Colonoscopy, 45–75, 15	-1.40	77.4	82.1	Dom.	-0.44	77.4	64.1	Dom.	0.62	66.5	50.5	Dom.	1.34	58.2	40.1	Dom.
**Colonoscopy, 45–75, 10** ^d^	**-1.31**	**83.6**	**87.8**	**71,700**	**-0.27**	**83.6**	**68.4**	**114,600**	**0.91**	**72.0**	**53.8**	**169,200**	**1.71**	**63.1**	**42.6**	**236,800**
Colonoscopy, 45–85, 10	-1.25	83.8	87.6	Dom.	-0.21	83.8	68.2	Dom.	0.95	72.1	53.5	Dom.	1.74	63.2	42.4	Dom.
**Colonoscopy, 45–75, 5**	**-0.56**	**90.1**	**89.9**	**354,000**	**0.56**	**90.1**	**69.0**	**1,222,700**	1.85	77.8	53.6	Dom.	2.74	68.3	41.7	Dom.
Colonoscopy, 45–80, 5	-0.50	90.4	89.7	Dom.	0.62	90.4	68.8	Dom.	1.92	78.0	53.3	Dom.	2.81	68.5	41.5	Dom.
Colonoscopy, 45–85, 5	-0.45	90.4	89.5	Dom.	0.67	90.4	68.5	Dom.	1.96	78.1	53.0	Dom.	2.84	68.5	41.3	Dom.
Esophageal cancer																
**No screening**	**0.0**	**0.0**	**0.0**	**Ref.**	**0.0**	**0.0**	**0.0**	**Ref.**	**0.0**	**0.0**	**0.0**	**Ref.**	**0.0**	**0.0**	**0.0**	**Ref.**
**Cytosponge, 60**	**976.2**	**32.8**	**32.4**	**30,200**	**1252.9**	**32.8**	**26.4**	**47,500**	**1312.2**	**32.1**	**24.3**	**53,900**	**1368.4**	**31.4**	**22.4**	**61,200**
**Endoscopy, 60** ^d^	**1618.3**	**40.5**	**38.4**	**106,300**	**1959.6**	**40.5**	**31.0**	**152,300**	**2032.6**	**39.6**	**28.5**	**173,200**	**2101.8**	**38.7**	**26.1**	**198,300**

Col: colonoscopy; ICER: incremental cost-effectiveness ratio; LY: Life year; QALY: quality-adjusted life-year; Ref: reference scenario.

**Fig 3:**
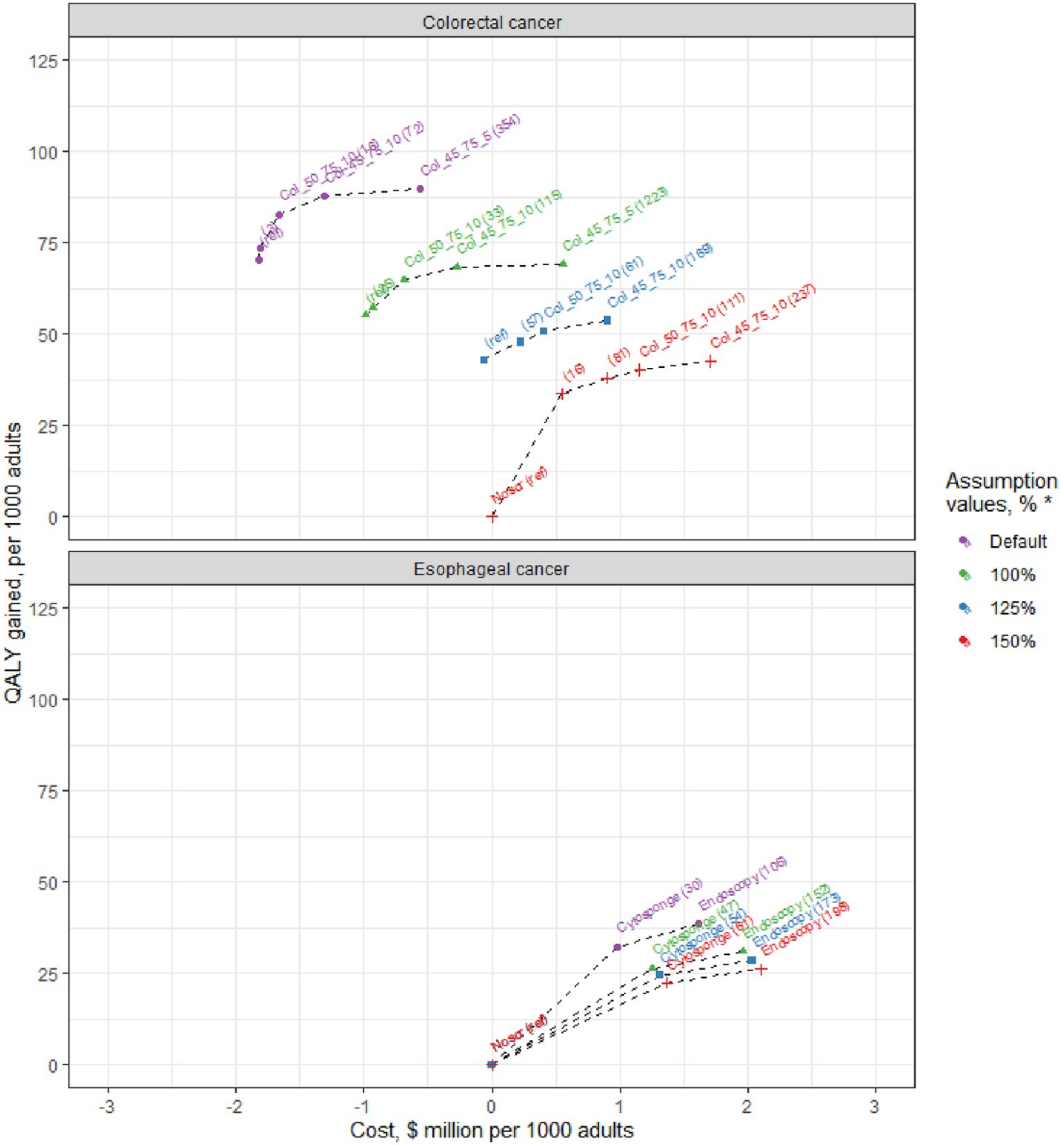
Impact of various assumed values for the future health-related costs, disutility and mortality, on the incremental cost-effectiveness of screening strategies for colorectal cancer and esophageal adenocarcinoma. ^a a^ Cost and QALY gained represented costs *vs*. no screening. The labels show the strategy, and in parentheses the incremental cost-effectiveness ratio ($1000). Assumptions for the health-related costs, disutility and mortality are presented as percentage of the estimated U.S. population averages. The default scenario assumed average mortality, and did not account for health-related costs and disutility.

^a^ Strategies are characterized as start age-stop age, interval, all in years. Bolded strategies are efficient in terms of the cost per QALY gained, and also presented in Fig 3.

^b^ No health-related costs and disutility for conditions other than the primary condition were included, but mortality from other causes was included.

^c^ Cost-effectiveness ratios were assessed incremental vs. the next less expensive efficient strategy.

^d.^ Recommended colonoscopy and upper endoscopy screening strategies.

### Incremental cost-effectiveness analysis

For CRC, when comparing all screening strategies under default assumptions for general health-related costs, disutility and mortality, there were five efficient colonoscopy screening strategies (Table 1, Fig 3). These include the recommended screening strategy of colonoscopy every 10 years from age 50 through 75 years, and the alternative starting from age 45 years. The ICER for these strategies was $15,600 and $71,700, respectively. With higher costs, disutility and mortality, the set of efficient strategies was similar. However, the ICER for the recommended strategy increased up to $111,100, and the ICER for the alternative strategy of colonoscopy every 10 years from age 45 increased to $236,800. Under “best-case” assumptions, the ICERs were $61,100 and $169,200, respectively.

For EAC, the ICERs for cytosponge and endoscopy screening under default assumptions for health-related costs, disutility and mortality were $30,200 and $106,300 per QALY gained, respectively (Table 1, Fig 3). Under extreme assumptions, ICERs approximately doubled. Best-estimate ICERs were $53,900 and $173,200.

In the sensitivity analysis without discounting of future outcomes, best-estimate ICERs for colonoscopy screening every 10 years starting at age 50 or 45 were $62,000 and $84,500, respectively. Best-estimate ICERs for EAC screening strategies were $39,200 and $95,700. In the analysis including only excess health-related expenses among patients with precancerous lesions or cancer, the best-estimate ICERs for the highlighted CRC screening strategies were $43,000 and $149,400, and the ICERs for the EAC screening strategies were $42,800 and $158,100.

## Discussion

In this exploratory study, we estimated the possible impact of health-related cost, disutility and mortality assumptions on the cost-effectiveness for two cancer prevention strategies, CRC and EAC screening. By contrasting scenarios with default assumptions *vs*. increased values in an established decision-analytic simulation model, we showed that ignoring these effects and costs may lead to misrepresentation of cost-effectiveness from a health sector perspective. For all three factors evaluated, we found a joint effect big enough to increase the ICERs of currently recommended strategies beyond common cost-effectiveness thresholds.

The relative importance of the specific model adjustments varied to some degree across the two models. For CRC, the currently recommended screening strategy was cost-saving compared to no screening under default assumptions of average mortality, and no accounting for costs or disutility for conditions other than CRC. This did not change with higher disutility or mortality rates. Higher costs, however, increased the ACER by the assumed per-capita cost amount by definition. For EAC, the disutility assumptions had the greatest individual impact on outcomes, and mortality assumptions the least. All factors combined nearly doubled the ACER for endoscopy screening up to >$80,000. Incremental cost-effectiveness ratios compared to other strategies were even more sensitive to these assumptions.

There is uncertainty regarding the relative risk of (co)morbidity among patients with precancerous lesions or cancer, i.e. those who may benefit from screening. Current EAC and CRC models often do not account for costs or disutility for other causes of morbidity, and assume an average life expectancy. Although implementing general population values already increased cost-effectiveness rates in our study *vs*. default assumptions, literature suggests that these patients may have an approximately 25% increased risk of other conditions. There is concern that those estimates may reflect selected patients. In a non-screening context, patients who have other conditions may be overrepresented among those receiving a colonoscopy or upper endoscopy. On the other hand, in SEER-Medicare linked data, CRC patients had a 43% increased risk of ≥2 comorbidities *vs*. non-cancer patients. Furthermore, the risk may increase for more *vs*. less advanced precancerous lesions [26]. More research is needed among average-risk screening populations, to inform these assumptions.

We assumed that health-related expenses, disutility and mortality are all proportional to the estimated relative risk of (co)morbidity. While this assumption may not be accurate, the population-based data used to inform our relative risk estimate did suggest similar order standardized incidence and mortality rates [21–23, 26]. A recent study from Germany also found a strong continuous relationship between perceived quality of life and mortality risk [27].

The impact of including future health-related costs or quality of life in cost-effectiveness estimates has been evaluated in previous studies [28–30]. In a recently published systematic review, authors evaluated the impact of medical costs and losses in quality of life due to other conditions on the cost-effectiveness of cancer screening within the U.S. context [30]. Consistent with our work, they found that considering the health-related expenses and disutility can substantially alter cost-effectiveness estimates for various screening programs. However, the study considered no potential excess risk of morbidity or mortality, which was the range were ICERS changed the most in our analysis.

We recognize that some of the assumptions in our analysis are controversial, and the main purpose of this analysis was to show the effect of different assumptions. For example, our base-case analysis included discounting of future outcomes, as well as decreasing age-related quality-of-life values. This is effectively double age-based weighting, which assigns relatively limited weight to outcomes at older ages. The sensitivity analyses suggested that these assumptions are consequential, with substantially lower cost-effectiveness ratios in case of no discounting. Also, the impact of the evaluated assumptions for cost may be smaller when considering compensatory influx of premiums and taxes collected during the life-years gained. Greater consensus regarding issues whether to include age-based weighting, lost productivity, all future costs, and associations in risks of primary and other conditions, would help increase comparability of future studies.

Our study had limitations. First, it was exploratory in its assumed ranges for the costs, disutility and mortality from conditions other than the one targeted by screening. Thus, the results cannot be used to directly support any clinical practice reforms. Further, we evaluated the cost-effectiveness outcomes from a healthcare sector perspective only, due to even greater uncertainty on indirect effects on productivity costs and other non-health related aspects, some of which may offset health-related costs.

Despite these limitations, our study has important implications. First, it indicates that health services researchers should be aware of the potential excess risk of comorbidities among patients with precancerous lesions or cancer *vs*. other patients, with substantive influence on cost-effectiveness outcomes. Better empiric evidence to inform this risk is warranted, as noted already. Further, in light of the suggested association between cancer and other outcomes, more research could be considered into what broader interventions would yield the greatest synergies in effect. Possibilities include both single interventions targeting multiple conditions (i.e. with “pleiotropic” effects), or portfolios of well-diversified interventions with minimal overlap in effects. Finally, adequate accounting for health-related costs, utility, and mortality may challenge accepted willingness-to-pay thresholds in different countries. If these factors systemically shift cost-effectiveness estimates for cancer screening and other interventions, it should be debated whether thresholds should also shift in response, or whether public health agencies should effectively become more selective by holding on to the same thresholds.

In conclusion, assumptions on the costs, disutility and mortality associated with other causes of morbidity may substantially alter the cost-effectiveness estimates for cancer screening. More empiric research and consensus are needed to guide assumptions in future research.

## Supporting information

S1 Appendix (DOCX)
