# Supplementary material for: Impact of assumptions on future costs, disutility and mortality in cost-effectiveness analysis; a model exploration
Source: PLoS One. 2021 Jul 12;16(7):e0253893. doi: 10.1371/journal.pone.0253893 (PMC8274850; doi:10.1371/journal.pone.0253893)
Supplement: S1 Appendix — (DOCX) [file pone.0253893.s001.docx]

**S1 Appendix**

This material is supplementary to:

Impact of assumptions on future costs, disutility and mortality in cost-effectiveness analysis; A model exploration.

**Table of Contents**

| Content | Page |
| --- | --- |
| S1 Fig. The MIcrosimulation SCreening Analysis (MISCAN) model | 2 |
| S1 Table. The impact inventory checklist for cost-effectiveness analysis conducted in this study | 3 |
| S2 Table. Costs and utility losses associated with colorectal cancer and esophageal adenocarcinoma screening and treatment | 6 |

Healthy

Precursor lesion

Preclinical cancer

Clinical cancer

Death from cancer

Other-cause death

*Opportunity for early diagnosis*

*Opportunity for cancer prevention*

**S1 Fig.** The MIcrosimulation SCreening Analysis (MISCAN) model

**S1 Table.** The impact inventory checklist for cost-effectiveness analysis conducted in this study.

| **Sector** | **Type of Impact**  (list category within each sector with unit of measure if relevant) | **Included in This Reference Case Analysis From…Perspective?** | | **Notes on Sources of Evidence** |
| --- | --- | --- | --- | --- |
|  |  | **Health Care Sector** | **Societal** |  |
| Formal Health Care Sector | | | | |
| Health | Health outcomes (effects) | | | |
|  | Longevity effects | ✓ |  |  |
|  | Health-related quality-of-life effects | ✓ |  |  |
|  | Other health effects (e.g., adverse events and secondary transmissions of infections) | ✓ |  |  |
|  | Medical costs | | | |
|  | Paid for by third-party payers | ✓ |  | Unrelated medical costs were explored in this paper |
|  | Paid for by patients out-of-pocket | ✓ |  |  |
|  | Future related medical costs (payers and patients) | ✓ |  |  |
|  | Future unrelated medical costs (payers and patients) | ✓ |  |  |
| Informal Health Care Sector | | | | |
| Health | Patient-time costs  Unpaid caregiver-time costs Transportation costs | NA  NA  NA |  |  |
| Non−Health Care Sectors (with examples of possible items) | | | | |
| Productivity | Labor market earnings lost  Cost of unpaid lost productivity due to illness Cost of uncompensated household production | NA  NA  NA |  |  |
| Consumption | Future consumption unrelated to health | NA |  |  |
| Social Services | Cost of social services as part of intervention | NA |  |  |
| Legal or  Criminal Justice | Number of crimes related to intervention  Cost of crimes related to intervention | NA  NA |  |  |
| Education | Impact of intervention on educational achievement of population | NA |  |  |
| Housing | Cost of intervention on home improvements (e.g. removing lead paint) | NA |  |  |
| Environment | Production of toxic waste pollution by intervention | NA |  |  |
| Other (specify) | Other impacts | NA |  |  |

**S2 Table.** Costs and utility losses associated with colorectal cancer (CRC) and esophageal adenocarcinoma (EAC) screening and treatment.

| **Variable** | **CRC model** | **EAC model** |
| --- | --- | --- |
| **Utility loss** | | |
| ***Per screening test*** |  |  |
| Colonoscopy with/without polypectomy | 2 days, 0.0055 | - |
| Endoscopy | - | 1 day, 0.0008 |
| ***Per treatment*** | - |  |
| Endoscopic eradication therapy | - | 16 days, 0.0131 |
| Radiofrequency ablation touch-up | - | 1 week, 0.0057 |
| ***Per complication*** |  |  |
| Stricture | - | 1 week, 0.0057 |
| Perforation | 2 weeks, 0.0384 | 8 weeks, 0.0460 |
| Bleeding | 2 weeks, 0.0384 | 1 week, 0.0057 |
| Serosal burn | 2 weeks, 0.0384 | - |
| ***Per life year with cancer care*** |  |  |
| Initial care | 0.12-0.70^a^ | 0.16-0.61^a^ |
| Continuing care | 0.05-0.70^a^ | 0.04-0.35^a^ |
| Terminal care, ending in cancer death | 0.70 | 0.04-0.61^a^ |
| **Costs, 2020 US $** | | |
| ***Per screening test*** |  |  |
| Colonoscopy with/without polypectomy | 938-1160 | - |
| Endoscopy | - | 814 |
| Cytosponge | - | 199 |
| ***Per treatment*** |  |  |
| Endoscopic eradication therapy | - | 6148 |
| Radiofrequency ablation touch-up | - | 1105 |
| ***Per complication*** |  |  |
| Stricture, Bleeding, Perforation | - | 1105-31,178 |
| ***Per life year with cancer care*** |  |  |
| Initial care | 35,093-78,767^a^ | 62,426-82,218^a^ |
| Continuing care | 2,771-12,679^a^ | 4,455 |
| Terminal care, ending in cancer death | 63,018-89,380^a^ | 70,654-93,047^a^ |
|  |  |  |

^a^ Depending on the cancer stage.
